# Supplementary material for: Modulating NLRP3 splicing with antisense oligonucleotides to control pathological inflammation
Source: Nucleic Acids Res. 2025 Nov 7;53(20):gkaf1116. doi: 10.1093/nar/gkaf1116 (PMC12596483; doi:10.1093/nar/gkaf1116)
Supplement: gkaf1116_Supplemental_File [file gkaf1116_supplemental_file.pdf]

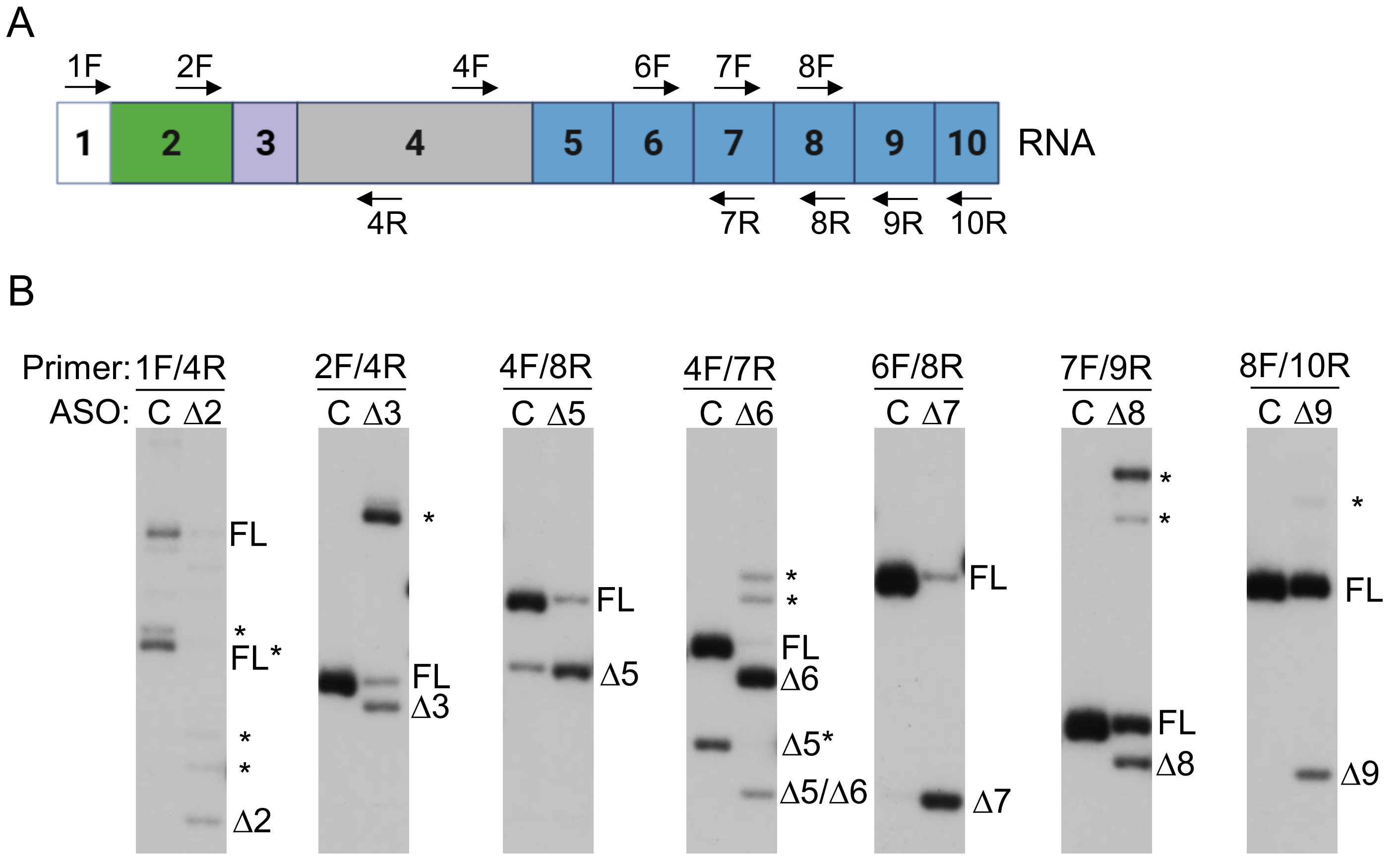

Supplemental Figure 1. Splice-switching ASOs modulate NLRP3 alternative splicing.
(A) Diagram of NLRP3 cDNA with PCR primer names, locations and direction indicated by arrows. **(B)** Radioactive RT-PCR gel from Figure 1, cropped to indicate the specific spliced products from each primer set and ASO treatment. FL denotes the full-length transcript, Δ indicates exon skipping of the corresponding exon. FL* represents a naturally occurring isoform that includes all coding exons, and Δ5* denotes a naturally occurring isoform lacking exon 5. Some ASOs also induced low levels of additional alternatively spliced products, marked with an asterisk (*).

Supplemental Figure 1

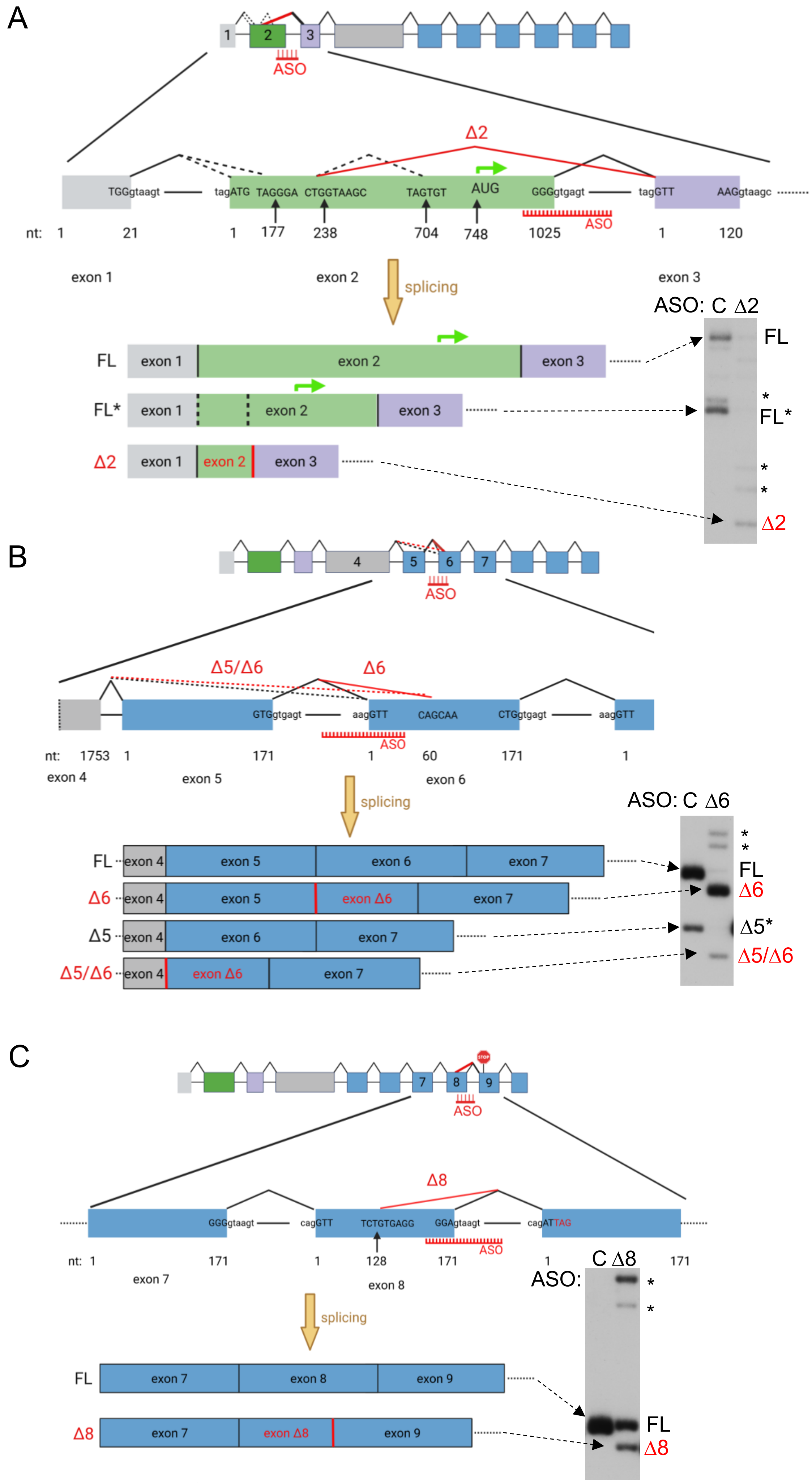

Supplemental Figure 2

Supplemental Figure 2. Graphical representation of ASO-induced splicing of exons 2, 6, and 8 from figure 1.

(A) Diagram of *NLRP3* exons 1–3 with ASO targeting exon 2 at the 5' splice site. AUG with green arrow indicates the start codon. Solid black lines represent constitutive splicing events and red solid lines indicate ASO-induced splicing. Black dashed lines represent a naturally occurring spliced isoform. Horizontal arrows point to the amplicons in the RT-PCR gel analysis that correspond to the spliced isoform diagrammed. The upper band corresponds to the full-length isoform (FL, exons 1–3). FL* refers to an isoform with an alternatively spliced exon 2. The ASO-induced Δ2 isoform is indicated in red. Minor splice products are denoted with an asterisk (*). **(B)** Diagram of exons 4–7 with ASO targeting exon 6 at the 3' splice site. Solid black lines represent constitutive splicing events and red solid line indicates ASO-induced exon skipping. Black dashed line represents a naturally occurring isoform lacking exon 5 (Δ5). Red dashed line depicts the naturally occurring isoform lacking both exon 5 and ASO-induced exon 6 skipping (Δ5/Δ6). In the RT-PCR gel, the upper band corresponds to the full-length isoform (FL, exons 4–7). Partial Δ6 isoform is labeled in red. Additional products include Δ5* (loss of exon 5) and Δ5/Δ6 (loss of both exons 5 and partial exon 6). Minor splice products are denoted with an asterisk (*). **(C)** Diagram of *NLRP3* exons 7–9 with ASO targeting exon 8 at the 5' splice site. Solid black lines represent constitutive splicing events and the red solid line indicates ASO-induced partial exon 8 skipping. In the RT-PCR gel, the upper band in the control sample corresponds to the full-length isoform (FL, exons 7–9). The Δ8 isoform (partial loss of exon 8) appears as the lower band (labeled red). Minor splice products are denoted with an asterisk (*).

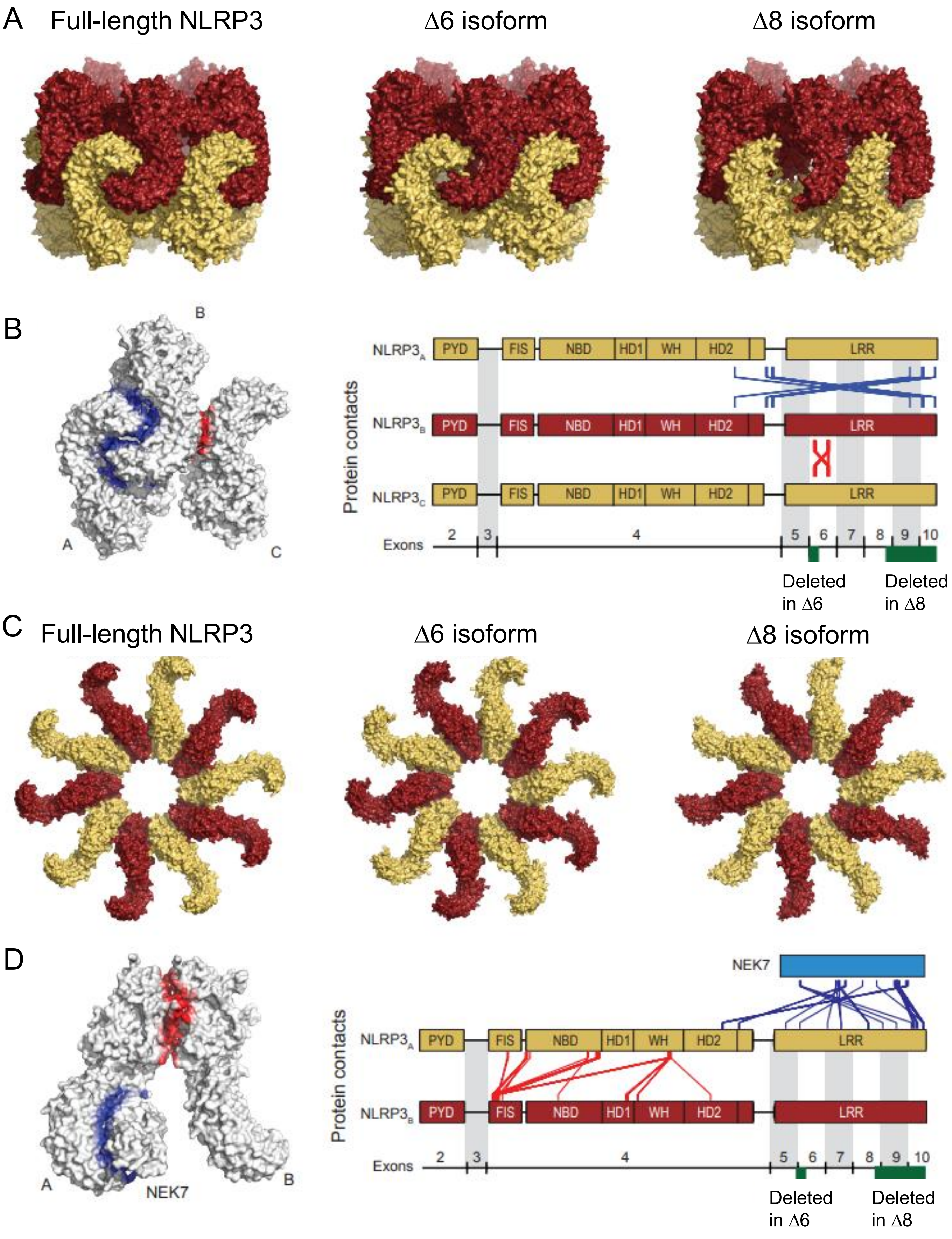

Supplemental Figure 3

Supplemental Figure 3. Modeling of NLRP3Δex6 and Δex8 proteins.

The NLRP3Δ6 and Δ8 isoforms are predicted to affect the stability of the inactive decamer cage assembly and potentially the NLRP3-NEK7 interaction in the active disk assembly. **(A)** Comparison of the inactive decameric cage form of full-length NLRP3 (left, PDB id 7pzc; (21)) and Alphafold2 models of the Δ6 and Δ8 isoforms superimposed onto the full-length structure. **(B)** Depiction of the two unique interaction surfaces of the decameric NLRP3 cage assembly between the concave (blue) and convex (red) faces of the LRR domains (left). The interactions mapped onto a linear diagram of the NLRP3 proteins reveals that the Δ6 isoform deletes a region of the LRR involved in the convex interaction (green box) and that the Δ8 isoform deletes most of the concave interaction region (green box). Both isoforms, but particularly the Δ8 isoform, are predicted to disrupt the stability of the decameric cage. In the case of the Δ6 isoform, the deletion could cause weakened convex interactions and alter the concave interactions by changing the spacing of the LRR repeats C-terminal to the deletion. **(C)** Comparison of the active disk form of full-length NLRP3 (left, PDB id 8ej4; (42)) and Alphafold2 models of the Δ6 and Δ8 isoforms superimposed onto the full-length structure. **(D)** Depiction of the two unique interaction surfaces of the active decameric NLRP3 disk assembly between adjacent NLRP3 monomers (red) and NEK7 (blue) (left). The interactions mapped onto NLRP3 protein linear diagrams suggests that both LRR deletions are unlikely to affect the decameric disk formation, as these interactions are mediated by the FIS and NACHT domains that are not affected in the Δ6 and Δ8 isoforms. In contrast, the Δ8 isoform eliminates many of the residues involved in the full-length LRR-NEK7 interaction (green box), suggesting a weaken interaction.

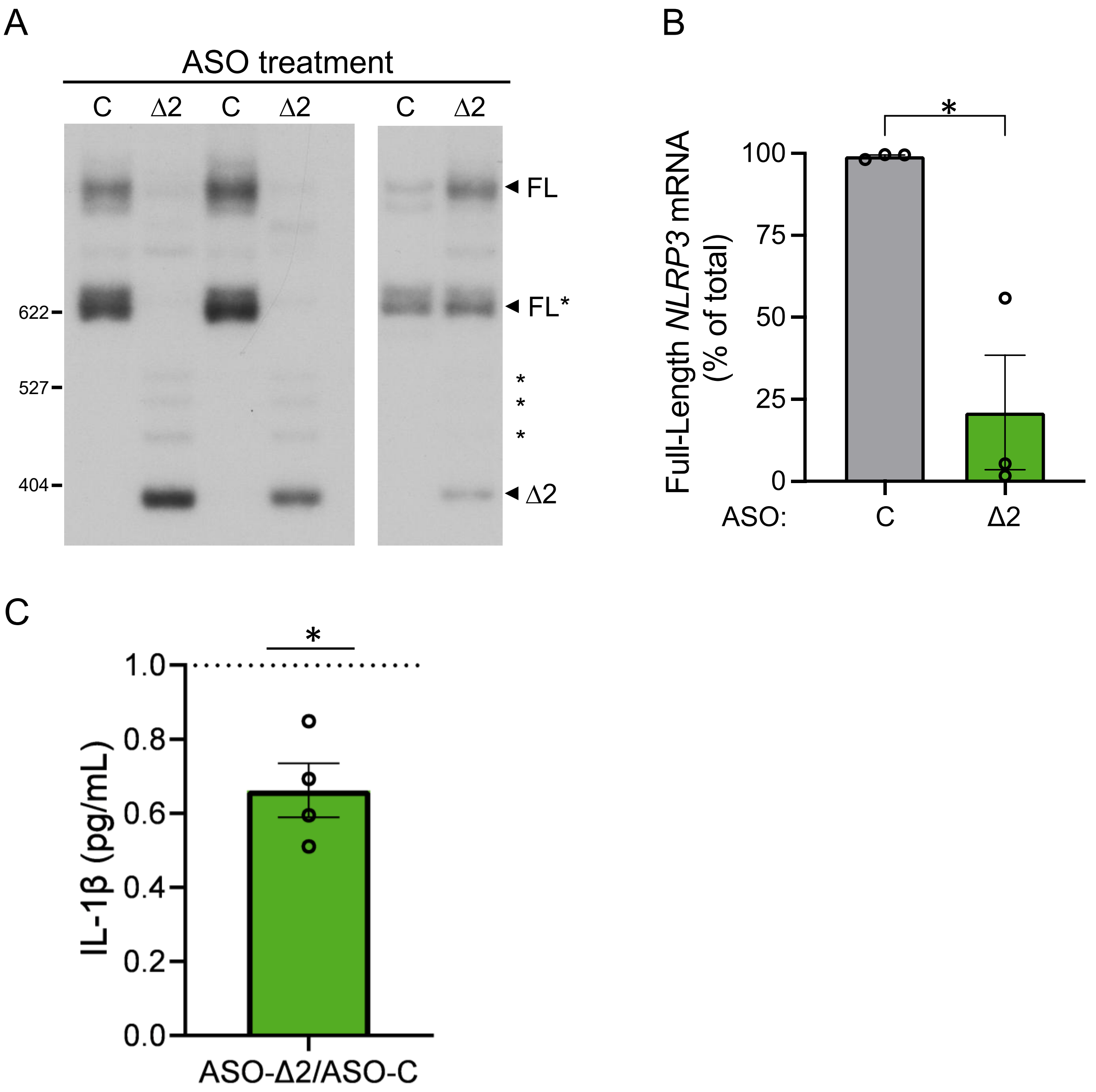

Supplemental Figure 4. ASO-Δ2 NLRP3 exon 2 splicing in human monocyte-derived macrophages (hMDM) from persons with CAPS.
(A) Radioactive RT-PCR analysis of NLRP3 isoform expression in NLRP3L353P/+ hMDM cells transfected with 40 μM of ASO-Δ2 or non-targeted ASO control (C) prior to activation with LPS. Other potential spliced products are denoted with asterisk (*). FL* denotes a naturally occurring full-length isoform. Products were amplified using a forward primer specific for NLRP3 exon 1 and a reverse primer in exon 4 and separated by PAGE. **(B)** Quantification of correctly spliced exons (full-length) from NLRP3 mRNA relative to the predominant ASO-Δ2 induced isoforms. Data are presented as mean ± SEM and analyzed by one sample t-test set to 1, *P<0.05; n=4 representing results of experiments using cells from three different patients, and cells from one patient analyzed in two independent experiments.

Supplemental Figure 4

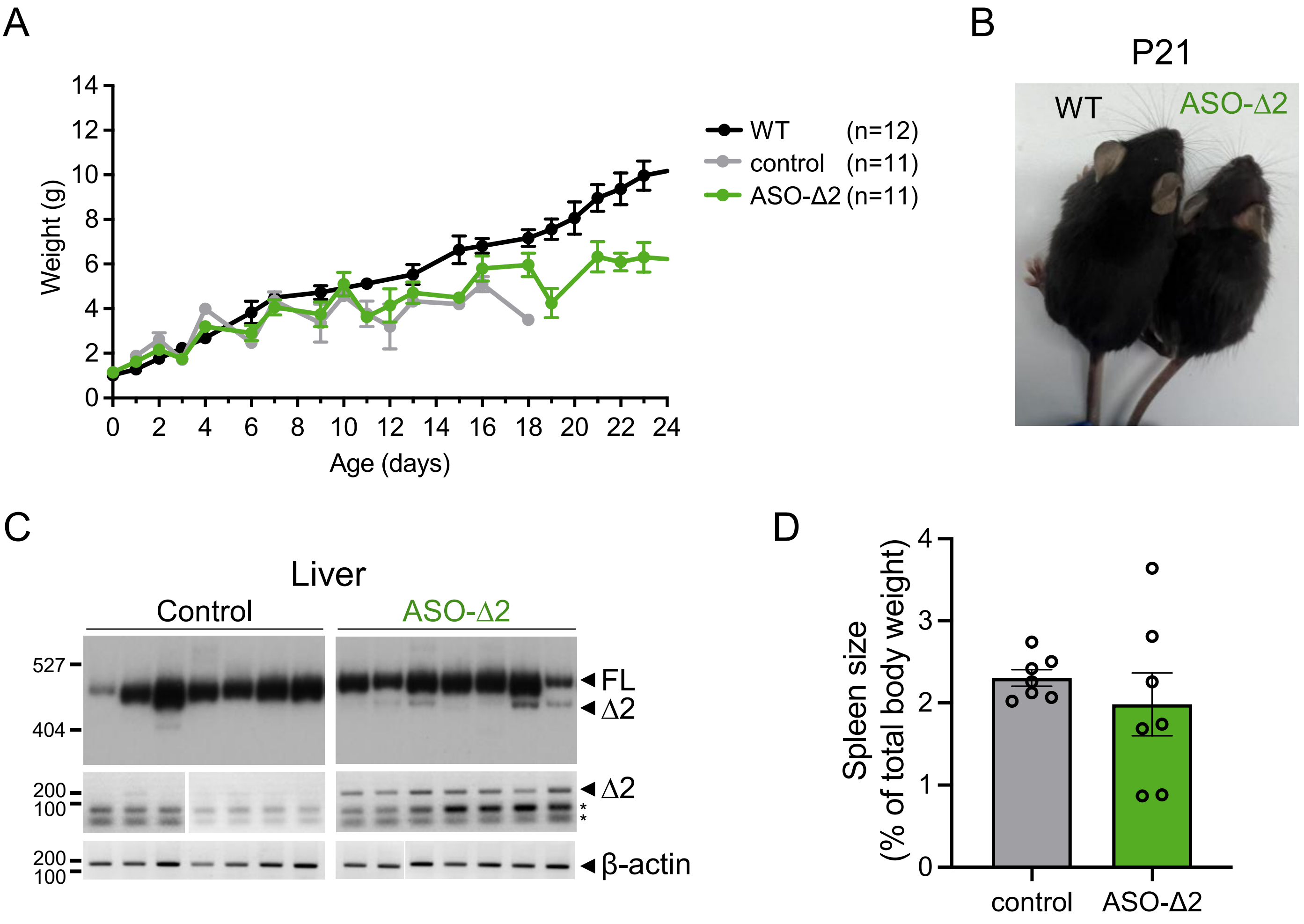

Supplemental Figure 5. Analysis of weights and splicing in ASO-Δ2-treated CAPS mice.

(A) Growth curve of ASO-Δ2 (n=11) and PBS control (n=11) treated Nlrp3D301N/+LysMCre+ mice and untreated wildtype (WT; n=12) mice. **(B)** Representative image of ASO-Δ2-treated Nlrp3D301N/+ LysMCre+ and WT mice at postnatal day 21 (P21). **(C)** (top gel) Splicing analysis of full-length Nlrp3 expression relative to partial Δ2 isoform in the liver using primers in exon 1 and 3. (middle gel) Amplification of the exon 2 skipped isoform only, using a primer that base pairs across the ASO induced cryptic 5' splice site in exon 2. (bottom gel) β-actin amplicon analyzed as a control of loading. Non-specific PCR products are denoted with asterisks (*). **(D)** Spleen size relative to total body weight of ASO-Δ2 and PBS control treated Nlrp3D301N/+LysMCre+ pups at P12/P13 (n=7). Data are mean ± SEM, unpaired t-test.

Supplemental Figure 5

Supplementary Table 1. Splice-switching Antisense Oligonucleotides and Primers

Primers	Sequence (5'-3')
hNLRP3ex1F	TTCCTGAGGCTGGCATCT
hNLRP3ex4R	TCTTGGTCTTGCCGATGGC
hNLRP3ex2F	CCGAGGGGTCAGACAGAGAA
hNLRP3ex4F	AGGCCGACACCTTGATA
hNLRP3ex7R	CCCAGTTTCTGCAGGTTAC
hNLRP3ex6F	CAGAAGCTGGTGGAGCTG
hNLRP3ex8R	GAGAGTGTTGCCTCGCAG
hNLRP3ex7F	CATTCCCTGACCAGACTCT
hNLRP3ex9R	CTCTGCTGTTTCAGCACTTC
hNLRP3ex8F	GTTGGTGAATTCTGGCCTTAC
hNLRP3ex10R	GAAGTGTTTCTAACGCAC
mNlrp3ex1F	TGGTCAGACACGAGTCCTG
mNlrp3ex3R	TGTCCTCCTGGCATACCA
mNlrp3ex2F	TGCGATCAACAGGCGAGAC
mNlrp3ex4R	CCGATGGTCAGGAGTTCATGCT
mNlrp3ex4F	GAGATCAACCTCTCTACCAG
mNlrp3ex7R	GAGCTGCTGTCTCACATC
mNlrp3ex6F	ATCAATGCTGCTTCGACATC
mNlrp3ex8R	GTAGATAGAGGTGTGTGAAG
mNlrp3ex7F	ATTGGTTCTGAGCTCCAACC
mNlrp3ex9R	CGCCAAGATCATTGTTGCCCAG
mNlrp3ex8F	TCCGGCCTTACTTCAATCTG
mNlrp3ex10R	TGGAGCGCTTCTAAGGCAC
mNlrp3ex2F-skipped isoform	TTTGGAAGATTACCCGCCCG
mNlrp3ex2ex3R- skipped isoform	TACACGTGTCATCTGTTGATCG
mβ-actinex2F	GGCTGTATTCCCCTCCATCG
mβ-actinex3R	CCAGTTGGTAACAATGCCATGT
**Oligos (PMO)**	**Sequence (5'-3')**
human NLRP3-201 e2i2	AGTCTTCCTTCCACTCACCCCACTT
human NLRP3-201 e3i3	GAAGCACCACCCCAGTCGCTTACCT
human NLRP3-201 e5i5	TTCACCAATCTAGGAATTAGAAGGA
human NLRP3-201 i5e6	GCGCCCCAACCTTCATGCCAAGAAC
human NLRP3-201 e7i7	ACACCCATGAAGACTTACCCCAGTT
human NLRP3-201 e8i8	AACCAAAGGACTTACTCCAACACCT
human NLRP3-201 e9i9	TGCAGAGCACACTCACCCCAGGTTC
mouse Nlrp3-202 e2i2	CCCTTCTGTTTACTCACTCCACTCT
mouse Nlrp3-202 e3i3	GCAGAAGCTTGACGCTTACCTTTCT
mouse Nlrp3-202 e5i5	CACACACAGACTTACCACAGTCTCT
mouse Nlrp3-202 e6i6	AGGTAGGCCAGACTCACCACAGTTT
mouse Nlrp3-202 i6e7	GCTCACCAACCTTCCATGCAGAGGG
mouse Nlrp3-202 e8i8	GGCAGCCCATGAACTCACTCCAGCA
mouse Nlrp3-202 e9i9	AGGCAAACCACACTCACTGTAGGCT
Non-targeted control	CCTCTTACCTCAGTTACAATTTATA
